# Development and cultural adaptation of the HOPE spiritual assessment tool to Swedish (HOPE-SE): Expert evaluation in specialized palliative care

**DOI:** 10.1017/S1478951526102533

**Published:** 2026-05-08

**Authors:** Johan Sundelöf, Bodil Holmberg, Christina Melin-Johansson

**Affiliations:** 1 Department of Public Health and Caring Sciences, Uppsala University, Uppsala, Sweden; 2 Department of Nursing Science, Sophiahemmet University, Stockholm, Sweden; 3 Department of Health Care Sciences/Nursing, Mid Sweden University, Östersund, Sweden

**Keywords:** HOPE tool, cultural adaptation, existential needs, spiritual care, palliative care

## Abstract

**Objectives:**

The HOPE spiritual assessment tool (HOPE tool), developed by Anandarajah and Hight, is a clinician-administered tool used to support the identification of patients’ existential, spiritual, and religious concerns. In Sweden, a foundational translation exists, but a culturally adapted version suited to a secular and multicultural context is lacking. This study aimed to develop a culturally adapted Swedish version (HOPE-SE) and assess its comprehensibility (face validity) and perceived relevance and coverage (content validity) among specialized palliative care professionals.

**Methods:**

Building on an earlier Swedish translation of the original 18-item HOPE, we conducted an observational cross-sectional development and expert evaluation study, in accordance with the STROBE (Strengthening the Reporting of Observational Studies in Epidemiology) guidelines, to simplify wording, improve item focus, streamline flow, and add prompts addressing non-religious existential concerns, resulting in a 16-item draft (HOPE-SE). Expert evaluation was conducted by 18 interdisciplinary experts in specialized palliative care (nurses, *n* = 6; physicians, *n* = 9; social workers, *n* = 3) who provided structured written feedback and participated in cognitive debriefing interviews. The written evaluation was summarized descriptively. The interviews were analyzed using descriptive content analysis of transcripts of the digitally recorded interviews.

**Results:**

Experts generally perceived HOPE-SE as understandable, acceptable, and clinically useful for initiating conversations about existential, spiritual, and religious concerns. Feedback led to minor wording refinements, clarification of potentially sensitive formulations, and the addition of a brief consent-based introduction to support timing and patient autonomy. The final HOPE-SE was approved by all experts and by the original HOPE author.

**Significance of results:**

HOPE-SE provides the first expert-reviewed Swedish conversation guide addressing existential, spiritual, and religious needs, intended to support spiritual history-taking in a multicultural healthcare context. Patient studies are needed to evaluate content validity and implementation in Swedish settings.

## Introduction

Spiritual and existential concerns are increasingly recognized as core components of holistic, person-centered care, particularly in palliative care settings (Puchalski et al. 2014; McCormack and McCance 2017; Batstone et al. 2020). A recent review concluded that spirituality – understood as meaning, connection, and transcendence – is associated with quality of life and coping in serious illness (Balboni et al. 2022). In secular Northern European contexts, spiritual and existential needs are often discussed across 3 overlapping domains: religious belief, spirituality (a connection beyond the material), and non-religious existential concerns such as meaning, mortality, and identity (Sand et al. 2008; Gijsberts et al. 2019). The European Association for Palliative Care describes spirituality as a dynamic dimension of human life related to meaning, purpose, and transcendence (Best et al. 2020). When unaddressed, these needs may be experienced as spiritual distress, for example, as fear, sadness, or loss of inner peace (Eshghi et al. 2023).

Despite increasing evidence for the value of spiritual care, spiritual and existential needs are frequently under-assessed in routine clinical practice (Balboni et al. 2022). Once identified, such needs can be supported through respectful listening and trustful relationships that enable existential conversations with patients and their next of kin (Lagerin et al. 2025). Several brief spiritual history-taking tools have been developed to support clinicians in this work (Lucchetti et al. 2013). Among these, the HOPE tool has been used internationally for more than 2 decades (Anandarajah and Hight 2001) and has been considered suitable for multicultural contexts (Blaber et al. 2015). A recent scoping review summarized HOPE’s continued clinical and research use across diverse settings (Sleeth et al. 2025).

A foundational Swedish translation of the original 18-item HOPE tool was previously piloted in specialized palliative care, and formative feedback suggested that refinement and cultural adaptation could improve clinical applicability in a secular and multicultural Swedish context, including clearer everyday language and prompts addressing non-religious existential concerns (Sundelöf et al. 2022). Sweden still lacks a peer-reviewed publication describing an expert-reviewed Swedish culturally adapted version of the HOPE tool suitable for exploring both religious, spiritual, and non-religious existential concerns. Therefore, the aim of this study was to develop a culturally adapted Swedish version (HOPE-SE) and assess its comprehensibility and perceived relevance and coverage (evidence for content validity) from the perspective of specialized palliative care professionals.

## Aim

This study aimed to develop a culturally adapted Swedish version (HOPE-SE) and assess its comprehensibility (face validity) and perceived relevance and coverage (content validity) among specialized palliative care professionals. The specific aims were to: (1) simplify wording to everyday Swedish; (2) avoid compound questions by ensuring 1 main concept per prompt; (3) reduce redundancy and keep the number of prompts feasible for routine clinical use; (4) include prompts for non-religious existential concerns; and (5) simplify administration by removing skip instructions.

## Methods

### Design

This observational cross-sectional study reports an expert evaluation of intended users as part of a multi-phase development program within the research initiative “Talk for life – conversations in palliative care.” The expert evaluation served as a pre-testing phase of the draft culturally adapted Swedish version, HOPE-SE, combining a structured written evaluation with cognitive debriefing interviews. The study is reported in accordance with the STROBE statement (von Elm et al. 2007).

### Development and cultural adaptation procedure

A foundational Swedish translation of the original 18-item HOPE tool was developed in 2012–2013 using forward and backward translation and review by a translation team of clinicians and researchers, guided by published cross-cultural adaptation procedures (Beaton et al. 2000; Wild et al. 2005; Sousa and Rojjanasrirat 2011). Building on that Swedish draft and formative pilot feedback of the original HOPE tool, indicating a need for refinement in a secular and multicultural context (Sundelöf et al. 2022), the authors (J.S., B.H., C.M.-J.) initiated a structured refinement in 2022. A key design goal was feasibility in routine clinical practice. This meant brief wording in everyday Swedish, a manageable number of prompts, and a format that could be used by interdisciplinary clinicians as a flexible conversation guide without requiring extensive specialist training. Local orientation and referral pathways may support implementation. In order to simplify administration, the skip instructions in the original version were removed. These steps resulted in a 16-item Swedish draft (HOPE-SE) organized in the same 4 domains as the original HOPE (H, O, P, and E). Table 1 summarizes the foundational Swedish translation of the HOPE tool.

**Table 1.** Foundational Swedish translation of the original 18-item HOPE tool (2012–2013) Table 1 long description.

Original version (English)	Swedish version
H: HOPE Sources of hope, meaning, comfort, strength, peace, love, and connection	H: HOPP Källor till hopp, välbefinnande, styrka, lugn inombords, kärlek och delaktighet
Q1. We have been discussing your support systems. I was wondering, what is there in your life that gives you internal support?	Vi har diskuterat dina källor till stöd och jag skulle vilja fråga dig; Vad i ditt liv skänker dig tröst och stöd i denna situation?
Q2. What are your sources of hope, strength, comfort, and peace?	Vilka är dina källor till hopp, styrka/energi, trygghet och inre ro/lugn?
Q3. What do you hold on to during difficult times?	Vad eller vilka kan du luta dig mot när det är svårt?
Q4. What sustains you and keeps you going?	Vad gör att du orkar och fortsätter trots den situation som är?
Q5. For some people, their religious or spiritual beliefs act as a source of comfort and strength in dealing with life’s ups and downs; is this true for you? If the answer is “‘Yes,’” go on to O and P questions. If the answer is “‘No,’” consider asking: Was it ever? If the answer is “‘Yes,’” ask: What changed?	För vissa personer kan religion eller tro tjäna som en källa till tröst och styrka när det gäller att hantera livets upp- och nedgångar, är det så för dig?
	Om svaret är “Ja”, gå vidare till O och P frågorna. Om svaret är “Nej”, överväg att fråga: Har det varit det tidigare?
	Om svaret på den andra frågan är “JA”, fråga: Vad ändrades?
O: Organized religion	O: Organiserad religion
Q6. Do you consider yourself part of an organized religion?	Betraktar du dig själv som religiös eller troende?
Q7. How important is this to you?	Hur viktigt är detta för dig?
Q8. What aspects of your religion are helpful and not so helpful to you?	Vilka delar av religionen hjälper dig och vilka delar av religionen är inte till så stort stöd eller har ingen större betydelse för dig?
Q9. Are you part of a religious or spiritual community? Does it help you? How?	Tillhör du en religiös församling? Hjälper det dig? Hur?
P: Personal spirituality/practices	P: Personlig andlighet/utövande
Q10. Do you have personal spiritual beliefs that are independent of organized religion? What are they?	Har du en personlig tro som inte är kopplad till en specifik religion? Hur ser den tron ut?
Q11. Do you believe in God? What kind of relationship do you have with God?	Tror du på Gud eller en högre livskraft? Vad tänker du att Gud eller denna livskraft är? Vilken relation har du till Gud?
Q12. What aspects of your spirituality or spiritual practices do you find most helpful to you personally? (e.g., prayer, meditation, reading scripture, attending religious services, listening to music, hiking, communing with nature)	Vilka delar av din andlighet eller ditt utövande hjälper dig mest (t.ex. bön, meditation, läsa skrifter, gå på religiösa möten)? Vad ger dig tröst och styrka i allmänhet? (exempelvis lyssna på familj, vänner, umgänge, underhållning, musik, göra utflykter, vara ute i naturen)?
E: Effects on medical care and end-of-life issues	E: Konsekvenser för medicinsk vård och frågor som rör vård i livets slutskede
Q13. Has being sick (or your current situation) affected your ability to do the things that usually help you spiritually? (Or affected your relationship with God?)	Har det faktum att du är sjuk (eller din nuvarande situation) påverkat din förmåga att utöva din religion eller det du brukar göra för att hitta stöd själsligt i tillvaron?
Q14. As a doctor, is there anything that I can do to help you access the resources that usually help you?	Som vårdpersonal, finns det någonting vi kan göra för att hjälpa dig att få tillgång till de resurser som normalt skänker dig andligt stöd?
Q15. Are you worried about any conflicts between your beliefs and your medical situation/care/decisions?	Är du orolig för någon konflikt mellan din tro och din medicinska situation/den medicinska vården/medicinska beslut?
Q16. Would it be helpful for you to speak to a clinical chaplain/community spiritual leader?	Skulle det vara till hjälp för dig att få tala med en sjukhuspräst eller en företrädare för den religion som du bekänner dig till? Skulle du vilja samtala med någon?
Q17. Are there any specific practices or restrictions I should know about in providing your medical care? (e.g., dietary restrictions, use of blood products)	Finns det något annat specifikt som jag behöver känna till som påverkar den medicinska vården för dig, t.ex. blodtransfusioner, kostrestriktioner?
Q18. If the patient is dying: How do your beliefs affect the kind of medical care you would like me to provide over the next few days/weeks/months?	Finns det något sätt du vill att din livssyn eller religion ska påverka den vård vi vill ge dig de kommande dagarna/veckorna/månaderna?

## Refinement and further development

### Participants and setting

In 2024/2025, an interdisciplinary expert panel (*N* = 18) was recruited from specialized palliative care services in Sweden, including permanent employee physicians, registered nurses, and counselors/social workers using purposeful sampling. Inclusion criteria were clinical experience in specialized palliative care and willingness to evaluate HOPE-SE from a clinical user perspective.

### Data collection

Participants completed a demographic web questionnaire covering information such as age, sex, profession, workplace, and experience in specialized palliative care. Data were also collected using a structured written evaluation form developed by the authors of this study, followed by evaluative interviews for cognitive debriefing (Wild et al. 2005; Brod et al. 2009). The written form included 15 statements: yes/no items and free-text comment fields focusing on comprehensibility, usability, and perceived coverage.

The interviews were facilitated by the authors and explored perceived relevance, appropriateness of wording and concepts, and suggestions for improvement. Interviews were collected through focus group interviews and individual interviews via secure digital audio recordings (Tong et al. 2007) and transcribed. Focus group interviews lasted 47–87 minutes (median 67 minutes), and individual interviews lasted 28–73 minutes (median 57 minutes).

### Data analysis

The analysis focused on recurring feedback and recommendations, consistent with standards for cultural validation and cognitive debriefing. The analysis aimed to identify actionable areas for refinement rather than quantify the frequency of individual suggestions; therefore, categories are presented descriptively using qualitative quantifiers (e.g., some, a few, several).

Yes/no responses from the written evaluation were described using numbers (*n*) and percentages (%); there were no missing data. Free-text answers were read and re-read to gain an overall understanding of the content, compared with the 16-item draft version of HOPE-SE, and used to inform further revision. All authors carried out the analysis in collaboration.

The interview data were analyzed using inductive qualitative descriptive analysis in accordance with established guidelines (Elo and Kyngäs 2008). Examples of the analysis of transcribed interviews are presented in Table 2. Relevant interview text was identified, extracted, and sorted into categories, supported by quotations. The tentative categories were discussed by all authors until consensus was reached.

**Table 2.** Examples of the analysis of transcribed interviews Table 2 long description.

Transcribed text	Sub-category	Main category
It (the instrument) creates an entry, an opening to inquire about existential needs based on clear questions.	Offers a structure for conversation	Possibilities
I am missing an important existential question concerning death itself, specifically what one feels and thinks about it.	Personal and emotional aspects are lacking	Barriers

To finalize the HOPE-SE, the authors independently reviewed and revised each item to ensure clarity, neutrality, and cultural appropriateness, with particular attention to non-emotive, inclusive language capturing religious, spiritual, and non-religious existential needs. Modifications were made to improve linguistic and cultural relevance, including deviations from the original phrasing when direct translation was not feasible; culturally specific or untranslatable terms were replaced with Swedish equivalents while preserving meaning. Findings and suggested refinements from the written evaluation and interviews were discussed by the authors, and a final HOPE-SE tool was developed based on consensus discussions.

## Results

### Demographic and clinical characteristics

Demographic and clinical characteristics of the participants are described in Table 3. Eighteen experts working in specialized palliative care participated: 6 nurses, 9 physicians, and 3 social workers. Most participants were aged 35–44 years (*n* = 8) and female (*n* = 15). Many had worked in their current workplace for 3–5 years and were employed in specialized palliative home care.

**Table 3.** Participant characteristics (*N* = 18) Table 3 long description.

Characteristic	*n*	%
Age, years		
35–44	8	44.4
45–54	5	27.8
55 or older	5	27.8
Sex		
Female	15	83.3
Male	3	16.7
Profession		
Physician	9	50.0
Registered nurse	6	33.3
Social worker	3	16.7
Workplace (all participants experienced in specialized palliative care)		
Specialized palliative home care	11	61.1
Specialized palliative in-hospital care	3	16.7
Specialized consultant team members	4	22.2
Experience in specialized palliative care		
4–6 months	2	11.1
7–12 months	4	22.2
1–2 years	0	0.0
3–5 years	5	27.8
6–9 years	3	16.7
10 years or more	4	22.2

### Written evaluation (structured questionnaire and free-text comments)

All 18 experts completed the structured written evaluation and provided illustrative free-text comments. Free-text comments and transcripts were grouped into 4 categories: perceived usefulness, wording and clarity, conceptual distinctions, and suggestions for missing content and preferred format (Table 4).

**Table 4.** Structured written evaluation of HOPE-SE (*N* = 18) Table 4 long description.

Question	Yes n (%)	No *n* (%)	Comments
1. Do you think it is possible to talk about existential needs based on the questions in the HOPE-SE conversation guide?	18 (100%)	0 (0)	“It creates an entrance/opening to ask about existential needs based on clear questions.” (P9)
2. Do you think it is possible to talk about religious needs based on the questions in the HOPE-SE conversation guide?	18 (100%)	0 (0)	“Yes, but the questions are very straightforward. I would rather use the [tool] as a support … and adapt them to what comes up in the conversation.” (P3)
3. Do you think it is possible to talk about spiritual needs based on the questions in the HOPE-SE conversation guide?	18 (100%)	0 (0)	“There may be a risk of confusing religion and spiritual needs. They are a bit intertwined, a bit similar.” (P1)
4. Are any of the questions of particular interest?	7 (38.9)	11 (61.1)	“Would like to see a question: What do you need from us (the caregivers) based on your faith/life view?” (P3)
5. Are any of the questions difficult to understand?	2 (11.1)	16 (88.9)	Abstract concepts could be cognitively demanding for some patients and situations (P2).
6. Are there any questions missing that are not asked in the conversation guide?	4 (22.2)	14 (77.8)	“Yes, under H: a question about thoughts of guilt and/or shame? Under O: Do you want help with religious ritual/ceremony?” (P1)
7. Are any question(s) irrelevant?	0 (0.0)	18 (100.0)	–
8. Do you think the conversation guide can be useful for identifying and meeting patients’ existential needs?	18 (100)	0 (0)	“Absolutely, it creates an entry point to open up to talk about these parts.” (P9)
9. Do you think the conversation guide can be useful for identifying what is important in assessing patients’ religious needs?	18 (100)	0 (0)	“Creates an entry point to bring it up and talk about it.” (P9)
10. Do you think the conversation guide can be useful for identifying and meeting what is important in assessing patients’ spiritual needs?	18 (100)	0 (0)	“Possibly put in the text, for example, in brackets (e.g. prayer, meditation, communion, etc.).” (P11)
11. Is it valuable to make assessments of patients’ existential needs?	18 (100)	0 (0)	“Very valuable in order to maintain a holistic view of the patient and move away from task-focused PC.” (P11)
12. Is it valuable to make assessments of patients’ religious needs?	18 (100)	0 (0)	“Important to include those who are believers, part of person-centered care. To be cared for and treated for who you are.” (P1)
13. Is it valuable to assess patients’ spiritual needs?	18 (100)	0 (0)	“It may be more common … to have spiritual needs over religion, as being at the end of life/severe illness can raise some existential thoughts.” (P1)
14. Is there anything you think should be changed or improved in the HOPE-SE conversation guide?	2 (11.1)	16 (88.9)	“Clearer that the paragraph under question 6 belongs to that question, it is unclear there.” (P9)
15. Do you have any other comments you would like to make?	2 (11.1)	16 (88.9)	“When and how often can this be used? Do you follow up? … Important for staff to think through the routine on the unit.” (P11)

#### Supporting conversations about existential, spiritual, and religious concerns

Across written responses, experts described HOPE-SE as understandable as well as potentially useful as structured support for initiating conversations that may otherwise be overlooked in clinical practice (Table 4). As one expert noted, it “creates an entrance/opening to ask about existential needs based on clear questions” (P9). Another emphasized that the guide may be most useful when used flexibly, as support that can be rephrased and adapted to what emerges in the conversation (P3).

#### Addressing spirituality and religiosity with sensitivity and relationship awareness

In the structured written evaluation, experts emphasized that spiritual and religious prompts may presuppose trust and continuity in the clinician-patient relationship. Several comments suggested that some questions – particularly those involving abstract concepts – may be cognitively demanding for patients, whereas more general care-related questions may feel less intrusive. Experts also noted that religious needs may become particularly visible when illness limits patients’ ability to practice their faith (e.g., attending services or performing rituals) (Table 4).

#### Refining wording and strengthening conceptual clarity

From the structured written evaluation analysis, a recurring theme was the need to refine formulations for clarity and cultural appropriateness. For example, several experts suggested rephrasing direct wording such as “Are you religious?” to a gentler alternative (e.g., “Do you have a faith?”). The Swedish term troende (“believer”) was described as potentially ambiguous, and experts noted that patients may not clearly distinguish between religious, spiritual, and existential concerns, indicating a need for brief clarification or clinician guidance and reflective competence to navigate these concepts. Experts also suggested avoiding compound questions (e.g., combining “hope” and “meaning”) and questioned whether some prompts might overlap (e.g., “inner peace”), potentially increasing redundancy.

Identifying missing content and preferred format, several experts requested clearer prompts addressing what patients perceive they need in terms of help and support, including practical support related to rituals or contact with a faith community when relevant (Table 4). Suggested additions also included prompts concerning guilt/shame and other existential concerns not otherwise captured in the core questions. Finally, experts expressed that binary yes/no formulations may restrict reflection and recommended more open-ended phrasing and a natural conversational flow. Some experts also preferred framing HOPE-SE explicitly as a flexible conversation guide rather than a screening instrument, allowing adaptation to patient readiness and clinical time constraints.

## Interview findings (cognitive debriefing)

All 18 experts participated in cognitive debriefing interviews, conducted as 4 focus groups (*n* = 13) and individual interviews (*n* = 5). Interview data were organized into 2 main categories – Possibilities and Barriers – with 6 sub-categories supported by quotations (Table 5). The results from the interviews identified new perspectives and confirmed the content that emerged in the free-text answers.

**Table 5.** Main categories and sub-categories from cognitive debriefing interviews with illustrative quotes (*N* = 18) Table 5 long description.

Main category	Sub-category	Illustrative quotes
Possibilities	Opens up for spiritual, existential, and religious conversations	With certain patients, it can be difficult to talk about things like this, but if this is a [tool] we always go through, it provides an entry point for discussion. Then it can really be something that can help. Then you get a legitimate reason to pose these questions. (P6)
Possibilities	Focus on the patient	Many patients say: I thought I was the only one with these questions, it felt like it was just me who had these crazy thoughts. Opening up for conversation allows them to put words to these thoughts. It can be a relief. (P7)
Possibilities	Offer a structure for conversation	The [tool] is good because it provides a structured way to suggest questions one can ask; I think that’s a strength, because it can easily feel a bit overwhelming. How am I supposed to ask any sensible questions in this advanced area when I’m not an expert? (P1)
Barriers	Spiritual, existential and religious questions presuppose a relationship	My experience is that many of these questions are quite private. I think it can be difficult to approach a person you may not know and start. It relies somewhat on some [tool] of trust or continuity to be able to approach some of these questions. At least if we expect to receive reasonable answers. (P10)
Barriers	Personal and emotional aspects are lacking	I know where to direct someone who has faith, but it’s harder to handle more general existential questions. Was my life meaningful? I find it difficult to cope with the feeling of injustice. Why did I get sick? Those kinds of questions that I would call existential but not necessarily religious. There is no obvious referral authority there. (P2)
Barriers	Unclear and vague questions	We are trying to find things that are hopeful and meaningful for the patients, but not all patients have spoken in these terms. What does meaning really mean? (P8)

### Possibilities

Experts described that HOPE-SE can open up conversations about existential, spiritual, and religious issues and provide legitimacy for raising sensitive topics, particularly when routine use reduces stigma and “demystifies” such discussions. HOPE-SE was also perceived as supporting a patient-centered approach by shifting attention from tasks to the patient’s broader situation and needs. Finally, the structured format was described as a strength by providing a manageable framework for clinicians who may feel uncertain about how to initiate existential conversations.

### Barriers

Experts emphasized that direct questions about religion or spirituality may feel private and can be challenging without a trusting relationship and sufficient competence to handle emotional reactions. Participants also perceived that the tool would benefit from a clearer prompt regarding what support the patient wants or needs, including practical support related to rituals or other valued practices. Finally, some questions were described as vague or conceptually demanding; experts recommended using clearer, open-ended formulations and allowing space for additional existential concerns not otherwise captured.

## Revisions and the final HOPE-SE

Based on expert feedback and the integration of all data, the authors made minor wording refinements, clarified potentially sensitive formulations, and added a brief consent-based introductory statement to support patient autonomy and appropriate timing. Suggestions for additional optional probes were discussed; only those considered feasible and broadly applicable were incorporated in the core tool, while others may be considered in future patient-based evaluation and implementation work. The final HOPE-SE was approved by all experts and by the original HOPE tool author (Table 6).

**Table 6.** The final culturally adapted Swedish version of the HOPE tool (HOPE-SE) Table 6 long description.

Modified English version	Swedish version
H: Sources of hope, meaning, comfort, strength, peace, love, and connection.	H: Källor till hopp, välbefinnande, styrka, lugn inombords, kärlek och delaktighet
H1: What is important to you in your current situation?	H1: Vad är viktigt för dig i den situation du befinner dig i?
H2: What or who can you lean on when it is difficult?	H2: Vad eller vilka kan du luta dig mot när det är svårt?
H3: What do you hope for?	H3: Vad hoppas du på?
H4: Is there anything that worries you?	H4: Finns det något som oroar dig?
H5: What gives you strength? What has helped you in difficult times before?	H5: Vad ger dig styrka? Vad har hjälpt dig tidigare när det varit svårt?
H6: What makes you happy?	H6: Vad gör dig glad?
H7: What makes you feel calm?	H7: Vad får dig att känna dig lugn?
H8: What gives you a sense of inner peace?	H8: Vad ger dig en känsla av lugn eller ro inombords?
H9: Do you feel that you receive the support you need?	H9: Känner du att du får det stöd du behöver?
O: Organized religion	O: Organiserad religion
O10: Do you consider yourself religious?	O10: Betraktar du dig själv som religiös eller troende?
O11: How important is your faith to you?	O11: (Om JA) Hur och på vilket sätt är detta viktigt för dig?
P: Personal spirituality/practices	P: Personlig andlighet/utövande
P12: Do you believe in a greater context (even if you do not identify as religious)?	P12: Tror du på ett större sammanhang (även om du inte är troende/religiös)?
P13: What aspects of your spirituality are helpful to you?	P13: (Om JA) Vilka delar av din andlighet är till hjälp för dig och på vilket sätt?
E: Effects on medical care	E: Konsekvenser eller betydelse för (effekt på) medicinska vårdens utformning (Effekt på)
E14: How has your illness or current situation affected what is important to you?	E14: Hur har din sjukdom eller det som har hänt påverkat din situation och det som är viktigt för dig?
E15: Is there anything you would like us to consider when planning your care based on your faith or worldview?	E15: Finns det något du vill att vi ska ta hänsyn till vid utformning av vården utifrån din tro/livssyn?
E16: Would you like to talk to someone about your situation (e.g., a social worker, spiritual/religious representative, psychologist)?	E16: Skulle du vilja samtala mer med någon utifrån din situation (till exempel kurator, andlig/religiös företrädare, psykolog)?

*Note:* Brief consent-based introduction (suggested): “HOPE-SE is a guide with questions that can help us talk about what gives you strength and what is important to you. We ask because this can matter for how we can best support you. You decide what you want to answer, and we can stop at any time.”

## Discussion

This study developed a culturally adapted Swedish version of the HOPE tool (HOPE-SE) and evaluated its comprehensibility, perceived relevance, and coverage through an expert panel in specialized palliative care. Across written feedback and cognitive debriefing interviews, experts described HOPE-SE as understandable and potentially useful for legitimizing conversations about existential, spiritual, and religious concerns that may otherwise remain unaddressed. At the same time, the findings emphasize that direct religious or spiritual prompts can be perceived as private and require sensitivity, trust, and appropriate timing in the clinical encounter.

Several brief spiritual history-taking tools are available for clinical practice, including FICA (Puchalski and Romer 2000), SPIRIT (Maugans 1996), and the recently developed I-SPIRIT (Steinhauser et al. 2024). Reviews of these tools emphasize that they should be applied as flexible guides rather than rigid screening instruments and that reported validation work is heterogeneous across settings (Lucchetti et al. 2013; Sleeth et al. 2025). In line with this literature, experts in our study valued HOPE-SE primarily as a clinician-administered conversation guide that offers structure without requiring scoring. The focus on hope, meaning, and sources of support may be particularly congruent with palliative care values and can help clinicians initiate and sustain person-centered dialogue (Blaber et al. 2015; Balboni et al. 2022).

Beyond explicitly religious prompts, in addition to items in the original HOPE tool, HOPE-SE includes non-religious existential themes such as current priorities/values, sources of support, hopes, worries, strength, and prior coping resources, positive affect, calm/inner peace, and how illness may affect what matters most. These themes overlap with domains included in spiritual-needs questionnaires used in palliative and chronic illness contexts, such as the Spiritual Needs Questionnaire, which contains existential and inner peace needs and items on fears/worries and meaning, and the Spiritual Needs Assessment for Patients, which includes a psychosocial domain alongside spiritual and religious needs (Sharma et al. 2012; Büssing 2021). By integrating these concerns into a brief clinician-administered conversation guide based on the original HOPE questions, HOPE-SE may be particularly well suited to multicultural contexts, including secular settings in which existential distress is not articulated in explicitly religious or spiritual terms.

The Swedish context is characterized by high secularization and increasing cultural diversity, which can make the concepts “spirituality” and “religion” both sensitive and ambiguous. Experts noted that patients may not distinguish between religious, spiritual, and existential concerns, and that clinicians may need brief guidance and reflective competence to navigate these concepts.

These observations echo earlier work describing clinicians’ uncertainty in spiritual care and the importance of relational competence, patient readiness, and consent when exploring existential concerns (Selman et al. 2018; Balboni et al. 2022). Overall, the expert panel perceived that the refinements using more everyday language and clearer prompts made HOPE-SE more accessible and usable as a practical conversation guide. Clinicians also described a substantial clinical need for a tool that can serve as a starting point for conversations, regardless of the patient’s belief system, when existential questions arise in multicultural, multi-faith, and secular contexts. In response to the expert feedback, we added a brief consent-based introduction to support acceptability and patient autonomy, reinforcing that HOPE-SE should be used when, and only if, the patient wishes to engage.

### Methodological considerations

Cross-cultural adaptation aims to achieve semantic, idiomatic, experiential, and conceptual equivalence so that prompts are understandable, culturally appropriate, and capture the intended concepts in the target context (Beaton et al. 2000; Wild et al. 2005; Sousa and Rojjanasrirat 2011). The modifications in HOPE-SE – simplifying language, avoiding compound prompts, and explicitly including non-religious existential concerns – were guided by these principles and by formative feedback from the Swedish pilot. In terms of measurement theory, this study provides initial evidence for face validity and for content-related aspects such as comprehensibility, relevance, and coverage from the perspective of intended clinical users. However, in line with Consensus-based Standards for the selection of health Measurement Instruments (COSMIN) guidance, full evaluation of content validity requires involvement of the target population (patients) to confirm relevance, comprehensibility, and comprehensiveness from the patient perspective (Terwee et al. 2018).

### Implementation considerations

HOPE-SE should be regarded as a conversation guide for spiritual history-taking rather than a tool for routine screening. A short consent-based introduction may help to clarify the purpose of the questions, support patient choice, and mitigate the risk that some prompts (especially religious ones) are experienced as intrusive. Clinicians should use HOPE-SE flexibly, prioritizing patient readiness and clinical context, and should be aware of local referral pathways (e.g., chaplaincy, counseling, psychology) when needs are identified.

### Strengths and limitations

Strengths include a structured multi-phase development process anchored in published cross-cultural adaptation procedures, clear refinement aims, an interdisciplinary expert panel, and triangulation across written feedback and cognitive debriefing interviews. The study approach, involving intended end-users in specialized palliative care, supported iterative, actionable revisions and enhanced credibility. The following limitations should be considered. The expert panel was purposively recruited and relatively small, and the findings may not represent perspectives in other Swedish healthcare settings. Because HOPE-SE is intended as a flexible clinician-administered conversation guide rather than a scored instrument, the study focused on comprehensibility, perceived relevance, and coverage; thus, other measurement properties (e.g., reliability, construct validity, and responsiveness) were not assessed. In addition, no widely accepted Swedish gold standard exists for criterion comparison. Importantly, this phase reflects clinician perspectives only. In line with COSMIN guidance, patient cognitive interviews and feasibility testing in routine care are required to confirm relevance, comprehensibility, and comprehensiveness from the patient perspective. Finally, interviews were conducted by the research team, which may have influenced responses, although the combination of written comments and investigator triangulation aimed to mitigate this risk.

## Conclusion

HOPE-SE is the first expert-reviewed Swedish conversation guide intended to support clinicians in exploring existential, spiritual, and religious concerns in a secular and multicultural healthcare context. Future studies should include patient involvement to fully evaluate content validity and explore implementation in Swedish healthcare settings.

## Data Availability

Data supporting the findings of this study are not publicly available due to the qualitative and potentially sensitive nature of the interview material but are available from the corresponding author upon reasonable request. De-identified excerpts may be shared to support verification of the analysis.

## Table 1 Long description

The table pairs each item from the English HOPE spiritual assessment tool with its Swedish translation. It is organized by the HOPE acronym: H (sources of hope and support) includes Q1–Q5; O (organized religion) includes Q6–Q9; P (personal spirituality/practices) includes Q10–Q12; and E (effects on medical care and end-of-life issues) includes Q13–Q18. Each row presents the English prompt on the left and the Swedish version on the right, including the section headers. One item (Q5) also includes conditional interviewer instructions about proceeding to later sections depending on the patient’s answer, and these instructions are translated as well. The Swedish wording often expands concepts (for example, adding “higher life force” alongside “God” and giving broader examples of supportive activities), while keeping the intent aligned with the English prompts. This is a translation mapping rather than numerical data, so comparisons are about phrasing and scope, not measured outcomes.

Navigate back to Table 1.

## Table 2 Long description

The table summarizes qualitative coding of two excerpts from transcribed interviews, linking each quote to a sub-category and a main category. One excerpt says the instrument creates an opening to ask clear questions about existential needs; it is coded as “Offers a structure for conversation” under the main category “Possibilities.” The other excerpt says an important existential question about death is missing, especially what people feel and think; it is coded as “Personal and emotional aspects are lacking” under “Barriers.” The entries contrast a strength (providing conversational structure) with a limitation (insufficient attention to emotional and personal reflections on death). Only two examples are included, so the categories illustrate coding rather than indicating frequency or prevalence.

Navigate back to Table 2.

## Table 3 Long description

The table summarizes participant demographics and professional background for 18 individuals, reporting counts (n) and percentages. The largest age group was 35–44 years (8; 44.4%), while 45–54 and 55+ were each 5 participants (27.8% each). Most participants were female (15; 83.3%) and 3 were male (16.7%). By profession, physicians were the largest group (9; 50.0%), followed by registered nurses (6; 33.3%) and social workers (3; 16.7%). All participants had experience in specialised palliative care; most worked in specialised palliative home care (11; 61.1%), with smaller shares in in-hospital care (3; 16.7%) and consultant teams (4; 22.2%). Experience levels were most commonly 3–5 years (5; 27.8%), with none reporting 1–2 years (0; 0.0%); 7–12 months and 10+ years each accounted for 4 participants (22.2%). Percentages may not sum to exactly 100% within sections due to rounding.

Navigate back to Table 3.

## Table 4 Long description

Responses from 18 participants rate a conversation guide across 15 yes/no evaluation questions, with optional qualitative comments. For questions 1–3 and 8–13, all participants answered Yes (18/18, 100%), indicating the guide supports talking about and assessing existential, religious, and spiritual needs and that such assessments are valuable. No one found any questions irrelevant (0/18 Yes; 18/18 No). Mixed feedback appears on whether any questions are of particular interest (7/18 Yes, 38.9%; 11/18 No, 61.1). Small minorities reported questions difficult to understand (2/18 Yes, 11.1%), missing questions (4/18 Yes, 22.2%), suggested improvements (2/18 Yes, 11.1%), or had other comments (2/18 Yes, 11.1%). Comments note the guide creates an “entry point,” but some questions may be too straightforward, could blur religion versus spirituality, and may benefit from clearer wording, examples (e.g., prayer/meditation), and added prompts (e.g., caregiver support needs, guilt/shame, rituals). Percentages reflect this single group of 18 respondents and may not generalize beyond the sample.

Navigate back to Table 4.

## Table 5 Long description

The table summarizes themes from 18 cognitive debriefing interviews, organized into two main categories—“Possibilities” and “Barriers”—each with sub-categories and illustrative participant quotes. Under Possibilities, participants said the tool can open spiritual, existential, and religious conversations by providing a legitimate entry point, keep attention on the patient by normalizing difficult thoughts and bringing relief, and offer a structured set of prompts that reduces feeling overwhelmed or unqualified. Under Barriers, participants noted that such questions can feel private and may require an established relationship, trust, or continuity to elicit meaningful answers. They also reported gaps in addressing personal and emotional existential concerns that are not explicitly religious, including uncertainty about where to refer patients for support. Another barrier was that some prompts may be unclear or vague (for example, what “meaning” means), making it harder to use consistently across patients. Quotes are examples of viewpoints and do not indicate how common each theme was among participants.

Navigate back to Table 5.

## Table 6 Long description

A two-column table pairs a modified English HOPE tool with its Swedish (HOPE-SE) wording. It is organized into four domains: H (sources of hope/meaning/strength and connection) with items H1–H9, O (organised religion) with O10–O11, P (personal spirituality/practices) with P12–P13, and E (effects on medical care) with E14–E16. Each row provides the English prompt and its Swedish equivalent, covering topics such as what is important now, sources of support, hopes and worries, strength, happiness, calm/inner peace, and whether needed support is received. The O and P sections include conditional follow-up wording in Swedish (e.g., “if yes”) for questions about being religious/believing and how spirituality helps. The E section links beliefs or worldview to care planning and offers options for further conversation with relevant professionals. The table presents translations rather than numerical results, so it supports comparison of phrasing and scope across languages rather than measuring outcomes.

Navigate back to Table 6.
